# Cyclodextrin Counteracts Coxsackievirus-Induced Cardiac Damage by Protecting Desmosome Integrity and Suppressing Proinflammatory Cytokine Expression

**DOI:** 10.3390/microorganisms13102294

**Published:** 2025-10-02

**Authors:** Guangze Zhao, Huifang M. Zhang, Grace J. Zhang, Wenli Yang, Christoph Küper, Bruce M. McManus, Decheng Yang

**Affiliations:** 1 Department of Pathology and Laboratory Medicine, University of British Columbia, Vancouver, BC V6T 1Z3, Canada; guangze.zhao@hli.ubc.ca (G.Z.); mary.zhang@hli.ubc.ca (H.M.Z.);; 2 Centre for Heart Lung Innovation, St. Paul’s Hospital, University of British Columbia, 1081 Burrard Street, Vancouver, BC V6Z 1Y6, Canada; 3 Department of Medicine, University of Pennsylvania Perelman School of Medicine, Philadelphia, PA 19104, USA; 4 MSH Medical School Hamburg, IMM Institute for Molecular Medicine, Medical University, 20457 Hamburg, Germany

**Keywords:** coxsackievirus B3, cyclodextrin, NFAT5, desmoplakin, proinflammatory cytokine, viral myocarditis

## Abstract

Nuclear factor of activated T cells 5 (NFAT5), an osmosensitive transcription factor, has been shown to protect against coxsackievirus B3 (CVB3)-induced myocarditis but is susceptible to cleavage by viral proteases. Identifying agents that upregulate NFAT5 may offer a novel antiviral strategy. Cyclodextrins, cyclic oligosaccharides that influence cellular osmolality, are promising candidates. In this study, we demonstrate that NFAT5 is critical for maintaining desmosomal integrity in cardiomyocytes. Cardiac-specific *Nfat5*-knockout mice showed a significant reduction in desmosomes, as observed by transmission electron microscopy. Furthermore, we identified desmoplakin (DSP), a structural desmosomal protein, as a direct transcriptional target of NFAT5, with reduced expression in *Nfat5*-knockout mouse hearts and *NFAT5*-knockdown HeLa cells. Notably, treatment with 5 mM cyclodextrin significantly upregulated NFAT5 expression with minimal cytotoxicity, restored DSP expression, and suppressed CVB3 replication by inhibiting viral RNA transcription, protein synthesis, and virion production. Additionally, cyclodextrin reduced mRNA levels of proinflammatory cytokines interleukin-1 beta and interleukin-8, indicating its potential role as an alleviator of excessive cytokine production. These findings identify NFAT5 as a key regulator of desmoplakin expression and prove cyclodextrin as a dual-functioning agent in counteracting cardiac damage through NFAT5-DSP-mediated protection of desmosome integrity and suppressing proinflammatory cytokine expression in CVB3-induced myocarditis.

## 1. Introduction

Intercalated disks (ICDs) are highly specialized cellular junctions that connect adjacent cardiomyocytes. ICDs are composed of three primary complexes: desmosomes, adherens junctions and gap junctions, each contributing uniquely to mechanical coupling and signal transduction in the heart [1]. Among these, desmosomes serve as the principal mediators of intercellular adhesion, ensuring that cardiomyocytes remain cohesively anchored within the myocardium. Mutations in desmosomal genes, such as those encoding γ-catenin, desmocollin and desmoglein, have been linked to a spectrum of inherited cardiac diseases, including arrhythmogenic right ventricular cardiomyopathy (ARVC), arrhythmogenic cardiomyopathy (ACM), and sudden cardiac death (SCD) [2,3,4].

Coxsackievirus B3 (CVB3), a member of the *Enterovirus* genus within the *Picornaviridae* family, is a primary etiological agent of viral myocarditis. It is a non-enveloped, positive-sense single-stranded RNA virus. Upon entering the cytoplasm, its genome is translated into a large polyprotein, which is then cleaved by viral proteases 2A and 3C into individual structural and nonstructural proteins. In addition to processing the viral polyprotein, proteases 2A and 3C also target numerous host cellular proteins during infection. Beyond eliciting a robust inflammatory response, CVB3 directly damages cardiomyocytes by inhibiting cellular transcription and translation and cleaving key structural and regulatory proteins [5]. Notably, CVB3 infection has been shown to disrupt desmosomal function, a process central to its pathogenesis. For instance, CVB3 upregulates miRNA-21, which downregulates desmin, a protein critical for desmosomal integrity, leading to the destabilization of the desmosomal complex [6]. Furthermore, CVB3 proteases cleave desmosomal proteins such as desmocollin-2 and desmoglein-2, promoting the degradation of γ-catenin and activating the Wnt signaling pathway [7]. These events collectively impair cardiomyocyte adhesion, contributing to the progression of cardiac injury.

In response to viral infection, host cells activate various transcription factors to mitigate damage and restore homeostasis. Among these, nuclear factor of activated T cells 5 (NFAT5) emerges as a key stress-responsive transcription factor. NFAT5 is upregulated under conditions of hypertonicity, heat shock, ischemia, and viral infection, where it regulates genes involved in cellular adaptation and immune responses [8]. However, CVB3 has evolved mechanisms to subvert host defenses, including the cleavage of NFAT5 by viral proteases during early infection [9]. This cleavage disrupts innate immune responses, facilitating viral replication and exacerbating cardiac damage. Nevertheless, NFAT5 has been implicated as a cardioprotective factor during CVB3 infection, suggesting that enhancing its expression could represent a promising therapeutic strategy to counteract viral-induced cardiac injury [10]. Given the critical role of desmosomes in maintaining cardiac integrity, we sought to investigate whether NFAT5 could influence desmosomal stability by regulating the expression of desmosomal proteins.

Cyclodextrins, the cyclic oligosaccharides known for their ability to modulate cellular osmolarity [11], have garnered attention for their potential as antiviral therapies [12]. These compounds exert their effects through diverse mechanisms, including inhibition of viral entry, enhancement of drug delivery, and modulation of host immune responses [13,14,15]. In the context of enteroviruses, cyclodextrins have been reported to inhibit viral replication by altering endosomal cholesterol distribution [16,17]. However, the potential interplay between cyclodextrins and NFAT5 remains unexplored. We hypothesize that cyclodextrins induce hypertonic stress, thereby upregulating NFAT5 expression and activating the transcription of target genes in desmosome.

In this study, we demonstrate that NFAT5 depletion reduces desmoplakin levels both in vivo and in vitro, and is associated with fewer desmosomes in *Nfat5*-knockout mouse hearts, a phenotype also observed in CVB3-infected wild-type mouse hearts. Moreover, we identify cyclodextrin as a potential antiviral agent against CVB3, demonstrating its capacity to inhibit viral genome replication, translation, and virion formation. Mechanistically, we see a dose-dependent relationship of cyclodextrin treatment leading to a progressive increase in NFAT5 expression, while mitigating CVB3-caused desmoplakin reduction in HeLa cells. This study elucidates the role of NFAT5 in preserving desmosomal integrity during CVB3 infection, and underscores the therapeutic potential of cyclodextrins in the treatment of viral myocarditis.

## 2. Materials and Methods

### 2.1. Mice, Cell Culture and Viral Infection

CVB3 (*Kandolf strain*) was propagated in HeLa cells (ATCC), and viral titers were determined using a plaque assay before infection. For in vivo experiments, all procedures were conducted in compliance with protocols approved by the Animal Care Committee of the Faculty of Medicine, University of British Columbia (protocol numbers: A18-0202 and A20-0065). Cardiac-specific *Nfat5*-knockout mice were generated as previously described [10]. Mice were infected with CVB3 at a plaque-forming unit (PFU) of 10^5^ via intraperitoneal injection, while the control group received an equivalent volume of saline. HeLa cells were cultured in Dulbecco’s Modified Eagle’s Medium (DMEM, #D6429, Sigma, St. Louis, MO, USA) supplemented with 10% fetal bovine serum (FBS, #A5256701 Thermo Fisher, Waltham, MA, USA), 2 mM glutamine, and antibiotics (100 μg/mL penicillin and 100 μg/mL streptomycin). Cells were infected with CVB3 at a multiplicity of infection (MOI) of 10 and collected at specific time points for analysis.

### 2.2. Cell Transfection

siRNAs targeting human NFAT5 (#sc-43968) were obtained from Santa Cruz Biotechnology (Dallas, TX, USA). HeLa cells were seeded in 6-well plates and incubated overnight at 37 °C. When the cells reached 30–40% confluency, they were treated with a transfection mixture composed of Opti-MEM™, siRNA, and Lipofectamine 2000 (#11668019, Life Technologies, Waltham, MA, USA) for 48 hours (h). Following transfection, cells were either harvested or subjected to further treatments.

### 2.3. Cyclodextrin Treatment

(2-Hydroxypropyl)-β-cyclodextrin (HPβCD) powder was purchased from ThermoFisher Scientific (#H31133.06) and dissolved in DMEM medium. HeLa cells were seeded in 6-well or 96-well plates and treated with HPβCD at different concentrations (0 mM, 0.1 mM, 0.5 mM, 1.0 mM, 5.0 mM and 10.0 mM) for 24 h. Then the cells were washed twice with phosphate-buffered saline (PBS), replenished with serum-free DMEM medium containing cyclodextrin, and infected with CVB3.

### 2.4. RNA Extraction and RT-qPCR

Total RNA was extracted from mouse tissues or cultured cells using the PureLink™ RNA Mini Kit (#12183018A, Invitrogen™, Thermo Fisher Scientific Inc., Waltham, MA, USA) following the manufacturer’s protocol. Subsequently, cDNA synthesis was performed using the extracted RNA and the LunaScript^®^ RT SuperMix Kit (#E3010L, New England Biolabs, Ipswich, MA, USA) to generate cDNAs. RT-qPCR reactions were carried out using Luna^®^ Universal qPCR Master Mix (#M3003L, New England Biolabs), cDNAs, and gene-specific primers. The expression levels of target genes were normalized to the endogenous control gene GAPDH. Each experiment included three technical replicates and five biological replicates to ensure reproducibility. The sequences of the primers used in this study are provided in Table S1.

### 2.5. Western Blotting

Cells were lysed with RIPA buffer containing protease inhibitors, while heart tissue samples were homogenized in RIPA buffer using a TissueLyser LT (Qiagen, Venlo, The Netherlands) and sonicated for 30 s at 40 Hz. After centrifugation, the supernatants were collected, and protein concentrations were determined using the Pierce™ BCA Protein Assay Kits (#A55864, Thermo Scientific™, Thermo Fisher Scientific Inc., Rockford, IL, USA). Equal amounts of protein were resolved by 4–12% SDS-PAGE and transferred onto nitrocellulose membranes (BioTrace NT, Pall Corporation, Oakland, CA, USA). The membranes were blocked with 5% skim milk in PBS and incubated overnight at 4 °C with primary antibodies. The following primary antibodies were used: monoclonal mouse anti-β-actin (Sigma-Aldrich), monoclonal mouse anti-VP1 (M47, Reutlingen, Germany), anti-NFAT5 (#21713-1-AP, Proteintech, Rosemont, IL, USA), anti-desmoplakin (#25318-1-AP, Proteintech), and anti-dsRNA (#76651, Cell Signaling, Danvers, MA, USA). After three washes, the membranes were incubated with HRP-conjugated secondary antibodies (goat anti-mouse or goat anti-rabbit IgG, Santa Cruz, Santa Cruz Biotechnology, Dallas, TX, USA). Protein signals were visualized using an enhanced chemiluminescence (ECL) detection system (ClarityTM Western ECL Substrate, Bio-Rad, Hercules, CA, USA) according to the manufacturer’s instructions.

### 2.6. Transmission Electron Microscopy (TEM)

Mouse hearts were perfused with saline, fixed overnight in 2.5% glutaraldehyde and 4% formaldehyde in 0.1 M sodium cacodylate buffer (pH 7.4), and rinsed with the same buffer. Tissues were then microwave-treated with 1% osmium tetroxide in 0.1 M sodium cacodylate buffer (pH 7.2) for 12 minutes (min) (PELCO 3441), dehydrated through an ethanol series, and embedded in Epon JEMBED812 resin. Ultrathin sections from Mid-LV were cut using a Leica EM UC7 ultramicrotome, stained with 2% uranyl acetate and lead citrate, and imaged using an FEI Tecnai Spirit electron microscope at 80 kV (UBC Bioimaging Facility).

### 2.7. Immunohistochemistry (IHC)

Mouse hearts were fixed in 4% paraformaldehyde-PBS and embedded in paraffin. Tissue sections were deparaffinized using CritiSolve and subjected to antigen retrieval by autoclaving in 1× citrate buffer (pH 6.0, Thermo Fisher) at 120 °C for 40 min. Slides were then treated with hydrogen peroxide for 5 min to block endogenous peroxidase activity. Next, staining was performed using the MACH 4 Universal HRP-Polymer Kit (Intermedico, Nea Kifisia, Greece). Slides were blocked with Background Punisher for 10 min and incubated overnight at 4 °C with primary antibodies diluted in TBS-PBS buffer (1% BSA, 0.15 M NaCl, 0.05 M Tris, pH 7.6). After that, slides were incubated with Polymer for 30 min at room temperature. Betazoid DAB Chromogen was applied for 5 min, and nuclei were counterstained with hematoxylin (Sigma, Gill No. 2) for approximately 5 min. Between each step, slides were washed with Tris-buffered saline (TBS) three times for 5 min each.

### 2.8. Immunofluorescence (IF)

Immunofluorescence was performed as previously described with minor modifications [10]. Cells grown on coverslips (#80826, Ibidi, Gräfelfing, Germany) were fixed in 4% paraformaldehyde for 20 min, washed three times with 0.1 M glycine in PBS (5 min each), and permeabilized with 0.1% Triton X-100 for 2 min. After three PBS washes, cells were blocked in 3% BSA for 1 h, then incubated overnight at 4 °C with specific primary antibodies. Following PBS washes, Alexa Fluor 488- or 594-conjugated secondary antibodies (Invitrogen) were applied, and nuclei were counterstained with DAPI (Sigma). Images were acquired on a Zeiss LSM 880 confocal microscope and processed using Zen Black/Blue (ZEN 3.12) software. We quantified fluorescence intensity using NIH ImageJ (ImageJ 1.54) with standardized imaging settings.

### 2.9. MTS Cell Viability Assay

HeLa cells were seeded in 96-well plates and treated with varying concentrations of cyclodextrin, followed by incubation at 37 °C for 24 h. Subsequently, 20 μL of combined MTS/PMS solution was added to each well containing 100 μL of cells in culture medium, according to the manufacturer’s instructions (CellTiter 96^®^ AQueous Non-Radioactive Cell Proliferation Assay, Promega, Madison, WI, USA). Absorbance was measured at 490 nm using an ELISA plate reader and all the assays were performed at least five times.

### 2.10. Viral Plaque Assay

CVB3-infected sample supernatants were serially diluted and added to HeLa cells in 6-well plates at 80–90% confluency. After a 1 h incubation, cells were washed twice with PBS, overlaid with 2 mL of 0.7% warm agar containing 1× complete DMEM supplemented with 10% FBS, and incubated for two days. Cells were then fixed with Carnoy’s fixative for 30 min and stained with 1% crystal violet. Viral plaques were counted manually, and titers were expressed as PFU/mL.

### 2.11. Statistical Analysis

Prism 10 (GraphPad Software, San Diego, CA, USA) was used for statistical analysis. Two-way ANOVA followed by Tukey’s test was used to determine differences among multiple comparisons. Student’s t-test with Welch’s correction was conducted to analyze the unpaired groups. Results are shown as mean ± standard error of mean (SEM) of three technical repeats, with *n* = 5 for biological repeats. A *p*-value less than 0.05 (indicated by *) was considered statistically significant. Additionally, nd = not significant; ** *p* < 0.01; *** *p* < 0.001; **** *p* < 0.0001.

## 3. Results

### 3.1. Deficiency of NFAT5 Destructs Desmoplakin and Desmosome Complex in Mouse Hearts

**Figure 1:**
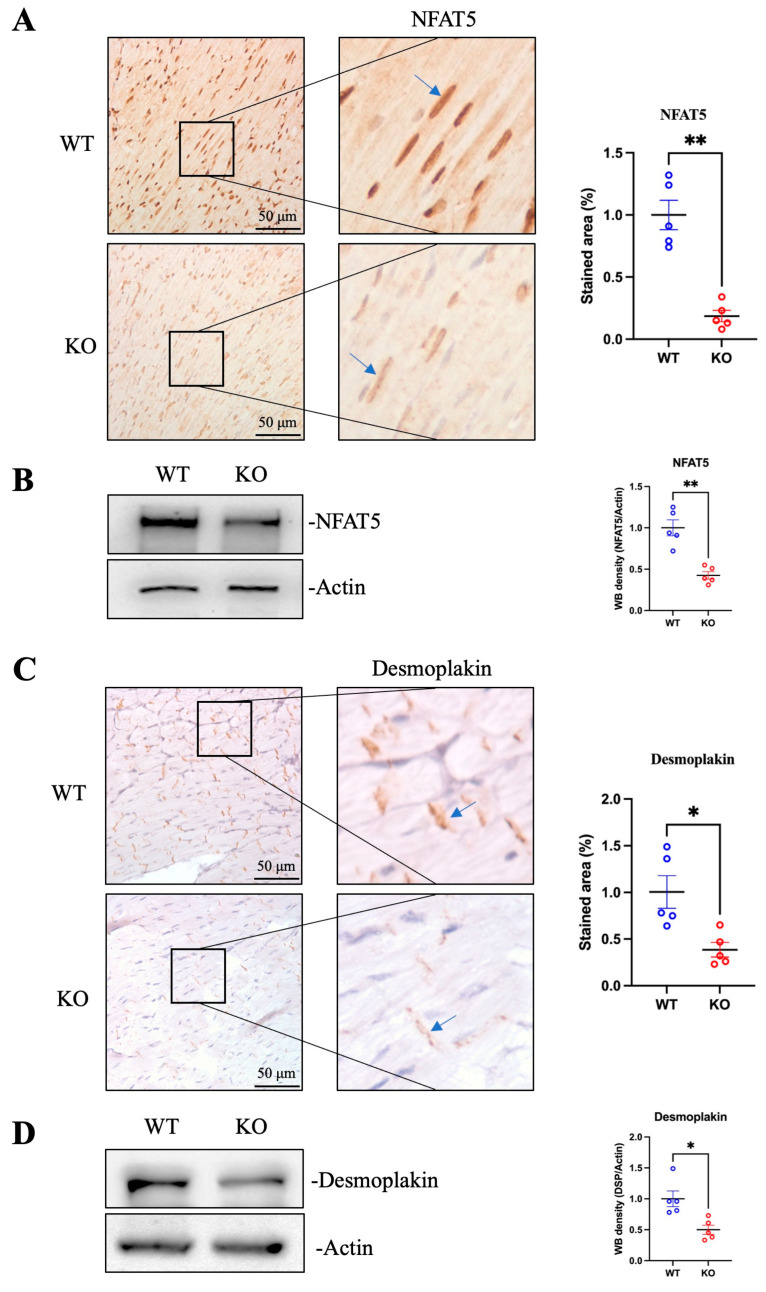

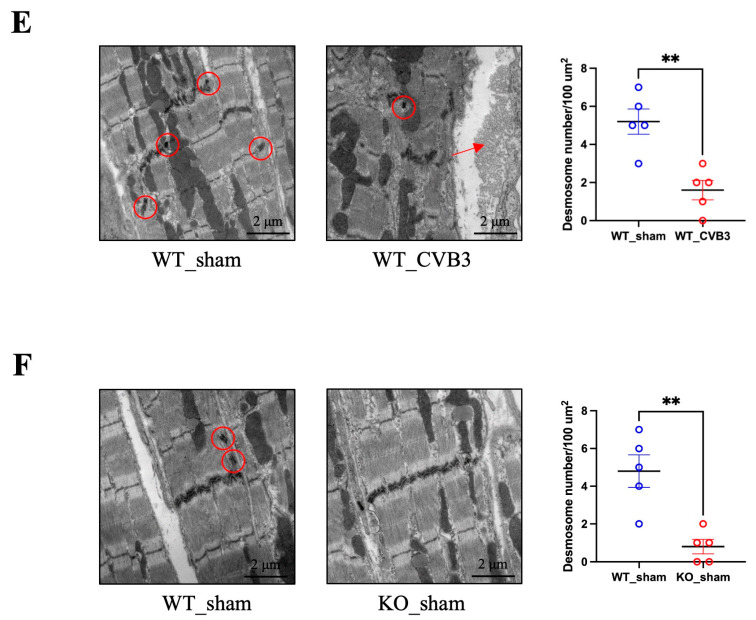
Cardiac-Specific *Nfat5*-knockout Reduces Desmoplakin and Disrupts Desmosomes in Mice. (**A**) Immunohistochemistry (IHC) staining and quantification of nuclear factor of activated T cells 5 (NFAT5) in heart tissues from wild-type (WT) and Nfat5-knockout (KO) mice. Brown staining indicates NFAT5 expression (blue arrows), and purple staining represents nuclei. (**B**) Detection of NFAT5 protein by Western blot from above heart tissues. Quantification of band density was conducted by ImageJ (*n* = 5). (**C**) IHC staining and quantification of desmoplakin (DSP) in heart tissues from WT and KO mice. Brown staining indicates DSP expression (blue arrows), and purple staining represents nuclei. Quantification of stained area was performed using ImageJ (*n* = 5). (**D**) Detection of DSP protein by Western blot from above heart tissues. (**E**) Transmission electron microscopy (TEM) images of intercalated disk (ICD) structures in hearts from Coxsackievirus B3 (CVB3)-infected mouse hearts. WT mice were either sham-infected with saline or infected with CVB3 for 7 days (*n* = 5 for each group). Heart samples were collected and processed for TEM to examine cardiomyocyte ultrastructure. Red circles highlight desmosomes, and red arrow indicates CVB3 particles. From each mouse, three randomly selected views (100 μm^2^ each) of heart sections were imaged and desmosome numbers were quantified and statistically analyzed. (**F**) TEM images of ICD structures in hearts from non-infected WT and KO mice (*n* = 5 for each group). Red circles highlight desmosomes. Statistical analysis was performed using Student’s *t*-test with Welch’s correction. Data are presented as mean ± SEM. * *p*< 0.05; ** *p* < 0.01.

To investigate whether the transcription factor NFAT5 regulates desmoplakin expression, we used a conditional cardiac-specific *Nfat5*-knockout mouse model [10]. Mice were injected with tamoxifen intraperitoneally at 4 weeks old to induce NFAT5 depletion, and hearts were subsequently harvested for analysis. IHC staining and Western blot confirmed the successful reduction in NFAT5 protein in the knockout hearts compared to wild-type controls (Figure 1A,B). Next, we examined the impact of NFAT5 deficiency on desmoplakin expression. IHC staining and Western blot of heart tissues from the same mouse cohort revealed a statistically significant decrease in desmoplakin protein levels, suggesting that NFAT5 depletion leads to a reduction in desmoplakin expression (Figure 1C,D).

Desmoplakin is a critical component of the desmosomal complex in the ICD structure, which is essential for cardiomyocyte connectivity. To determine whether the reduction in desmoplakin affects the structural integrity of the ICD, we employed TEM to examine cardiomyocyte ultrastructure. In hearts infected with CVB3, a cardiotropic virus known to cleave and degrade NFAT5 through its proteases [9], we observed a decrease in the number of desmosomes (Figure 1E). Similarly, TEM analysis of *Nfat5*-knockout hearts revealed a nearly complete loss of desmosomal structures compared to wild-type hearts (Figure 1F). These findings suggest that the reduction in NFAT5, due to either CVB3 infection or genetic knockout, disrupts desmosomal integrity in the ICD. This effect appears to be mediated, at least in part, by the downregulation of desmoplakin, which is regulated by NFAT5.

### 3.2. Desmoplakin Is a Direct Transcriptional Target of NFAT5

**Figure 2:**
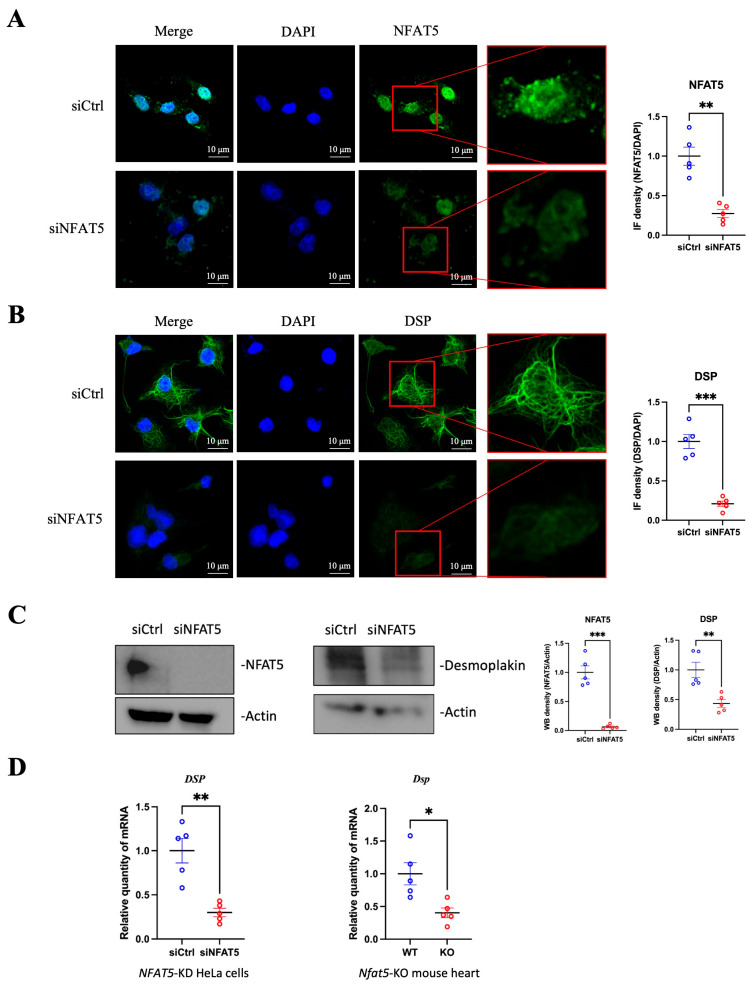
*DSP* is a Direct Transcriptional Target of NFAT5. (**A**) Immunofluorescence (IF) of NFAT5 in HeLa cells transfected with NFAT5-specific siRNA (siNFAT5) or scrambled control siRNA (siCtrl), followed by IF of NFAT5 protein. Green fluorescence indicates NFAT5, and blue fluorescence (DAPI) marks nuclei. Quantification of immunofluorescence density was performed by ImageJ. (**B**) IF of DSP in HeLa cells transfected with siNFAT5 and subjected to IF for desmoplakin. Green fluorescence indicates desmoplakin protein, and blue fluorescence indicates nuclei. (**C**) Western blot analyses of NFAT5 and DSP proteins using HeLa cells transfected with siNFAT5 or siCtrl. Quantification of Western blot density was performed by ImageJ. (**D**) Determination of *DSP* mRNA levels by RT-qPCR using total RNA extracted from HeLa cells transfected with siNFAT5 (left) or Nfat5-knockout mouse heart tissues (right). Statistical analysis was performed as in Figure 1. Data are presented as mean ± SEM, *n* = 5. * *p* < 0.05; ** *p* < 0.01; *** *p* < 0.001.

To further unravel the role of NFAT5 in regulating desmoplakin expression, we performed in vitro experiments by transfecting HeLa cells with either NFAT5-specific siRNA (siNFAT5) or scrambled control siRNA (siCtrl). IF staining demonstrated a remarkable reduction in NFAT5 protein levels in siNFAT5-transfected cells compared to controls, confirming efficient knockdown of NFAT5 (Figure 2A). Subsequent IF staining for desmoplakin showed a significant decrease in its expression following NFAT5 knockdown (Figure 2B). Western blot analysis corroborated the reduction in desmoplakin protein levels in *NFAT5*-knockdown cells (Figure 2C).

To determine whether this regulation occurs at the transcriptional level, we analyzed *DSP* mRNA levels by RT-qPCR and observed a significant decrease in *DSP* transcripts upon *NFAT5*-knockdown. This finding was further validated in vivo, where *Dsp* mRNA expression was significantly lower in the hearts of *Nfat5*-knockout mice compared to wild-type controls (Figure 2D). To rule out the potential mechanism underlying how NFAT5 regulates *DSP* mRNA stability, we predicted NFAT5 target sites by screening for NFAT5 binding motifs within the promoter region of the *DSP* gene, defined as 5000 base-pair (bp) upstream to 1000 bp downstream of the transcription start site (TSS). Our analysis identified a potential NFAT5 binding site located 596 bp upstream of the *DSP* TSS (Figure S1). Collectively, these results demonstrate that NFAT5 positively regulates desmoplakin expression, and its depletion leads to reduced desmoplakin levels at both the protein and mRNA levels, indicating that desmoplakin is a transcriptional target of NFAT5.

### 3.3. Determination of Optimal Cyclodextrin Concentration in HeLa Cells for Antiviral Evaluation

**Figure 3:**
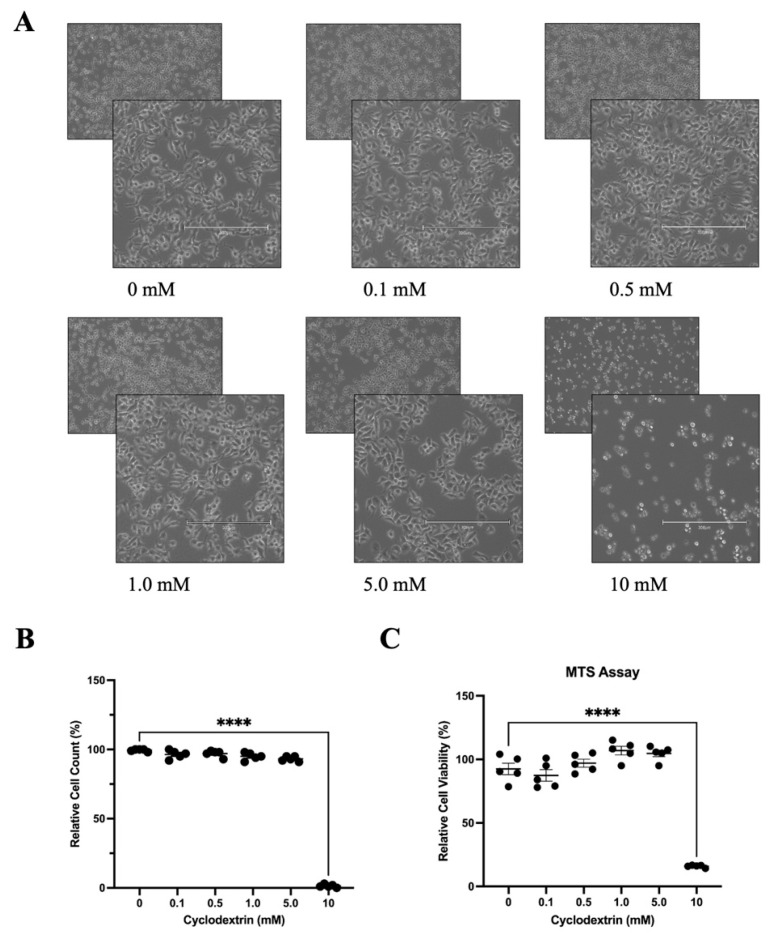
Determination of Optimal HPβCD Concentration in HeLa Cells for Antiviral Evaluation. (**A**) Morphology of HeLa cells under treatment of (2-Hydroxypropyl)-β-Cyclodextrin (HPβCD). Cells were treated with varying concentrations (0, 0.1, 0.5, 1.0, 5.0, and 10 mM) of HPβCD for 24 h and then visualized under an EVOS M5000 microscope, scale bar = 300 μM. (**B**) Counted numbers of healthy HeLa cells under HPβCD treatment. Cell viability was evaluated by counting dead cells in five randomly selected fields per image. (**C**) MTS cell viability assay of HeLa cells under HPβCD treatment. Statistical analysis was performed as in Figure 1. Data are presented as mean ± SEM (*n* = 5). **** *p* < 0.0001.

Cyclodextrin is a broad-spectrum component used in various drug research and antiviral studies [13]. Particularly, its nature in modulating cellular osmolality implies its potential in inducing expression of osmosensitive transcription factor NFAT5 [11]. Verifying this speculation may provide molecular evidence for the antiviral activity of cyclodextrin on the upregulation of NFAT5. To this end, we first determined the optimal concentration of cyclodextrin for antiviral evaluation. HeLa cells were treated with different concentrations of HPβCD (0, 0.1, 0.5, 1.0, 5.0, and 10 mM) for 24 h. Morphological examination using an EVOS M5000 microscope (Invitrogen, Waltham, MA, USA) revealed no noticeable changes in cell appearance or viability at concentrations up to 5.0 mM (Figure 3A,B). By MTS assay we further confirmed that 5 mM HPβCD does not significantly affect cell viability compared to untreated control cells, indicating that this is a non-cytotoxic concentration (Figure 3C). In contrast, treatment with 10 mM HPβCD caused extensive cell death, with cells appearing rounded, detached, and non-viable. These results suggest that low to moderate concentrations of HPβCD (≤5.0 mM) are well tolerated by HeLa cells, whereas high concentrations (≥10 mM) are cytotoxic and lead to cell death.

### 3.4. Antiviral Effect of Cyclodextrin Is Associated with NFAT5 Upregulation

**Figure 4:**
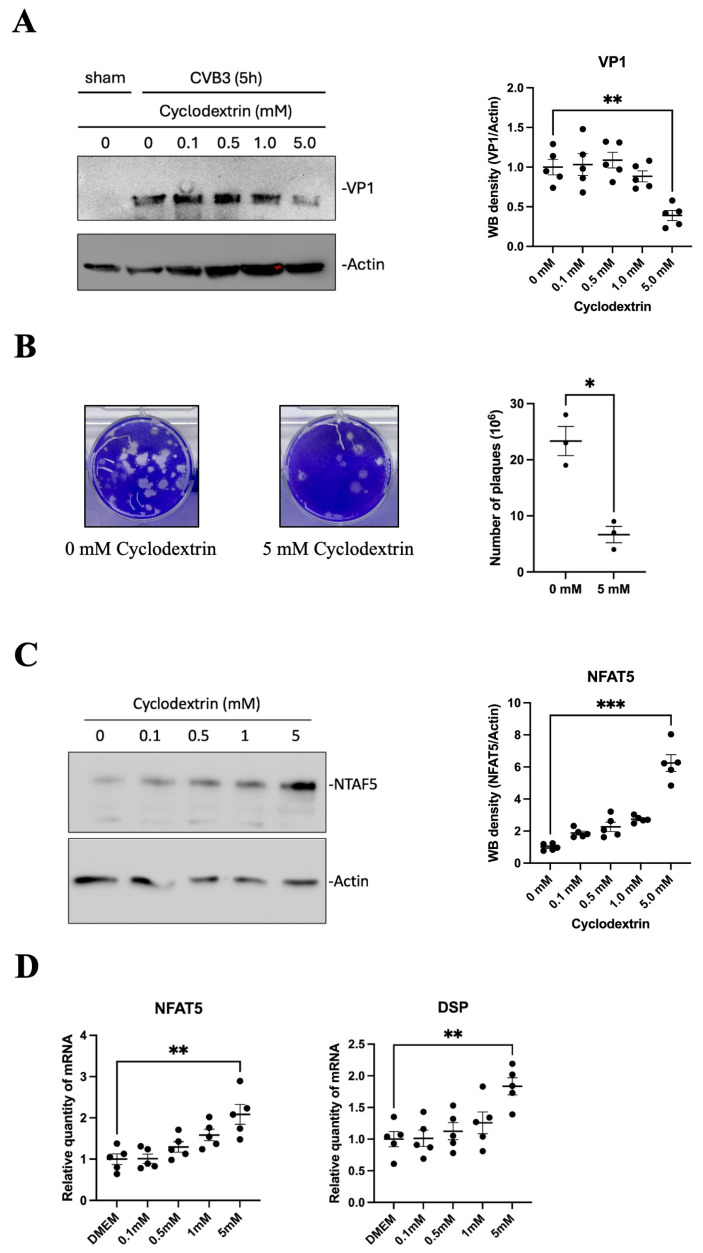
HPβCD Inhibits CVB3 Replication and Induces NFAT5 Expression. (**A**) Western blot analysis of CVB3 VP1 protein levels after HeLa cells were pretreated with HPβCD (0–5.0 mM) for 24 h and subsequently infected with CVB3 (MOI = 10) for 5 h. Quantification of Western blot analyses was performed by ImageJ. (**B**) Plaque assay to quantify infectious viral particles. Culture supernatants were collected from infected cells in (**A**) and serially diluted to 10^−6^. (**C**) Western blot analysis of NFAT5 protein levels from cell lysates in (**A**). (**D**) Quantification of *NFAT5* and *DSP* mRNA levels by RT-qPCR using total RNA extracted from the same cell lysates in (**A**). Statistical analysis was performed as in Figure 1. Data are presented as mean ± SEM (*n* = 5). * *p* < 0.05; ** *p* < 0.01; *** *p* < 0.001.

Cyclodextrins, which have hydrophobic cavities, have been shown to suppress CVB3 infection by extracting cholesterol from the plasma membrane [18], thus blocking viral entry [19]. To further determine whether cyclodextrin inhibits CVB3 infection at the replication level after entry, we treated HeLa cells with HPβCD at a 5 mM concentration for 24 h, and then infected them with CVB3 (MOI = 10) for 5 h. Western blot analysis showed a reduction in VP1 protein expression—a viral capsid protein and marker of CVB3 replication—indicating that HPβCD suppresses viral protein synthesis (Figure 4A). Supporting this finding, plaque assays also demonstrated a significant reduction in the number of infectious viral particles following HPβCD treatment compared to untreated controls (Figure 4B).

To further investigate whether cyclodextrin suppresses CVB3 replication through the upregulation of NFAT5, HeLa cells were pretreated with increasing concentrations of HPβCD (0, 0.1, 0.5, 1, and 5 mM) for 24 h. Cell lysates were then collected and analyzed by Western blot to assess NFAT5 protein levels (Figure 4C). Notably, HPβCD treatment significantly increased NFAT5 protein expression at 5 mM compared to untreated controls. Consistent with this result, RT-qPCR analysis revealed a dose-dependent increase in *NFAT5* mRNA levels, with 5 mM HPβCD significantly enhancing *NFAT5* transcription. Similarly, *DSP* mRNA levels followed the same trend, showing significant upregulation with 5 mM HPβCD treatment (Figure 4D).

### 3.5. Cyclodextrin Treatment Restores Desmoplakin Levels Reduced by CVB3-Caused NFAT5 Deficiency

**Figure 5:**
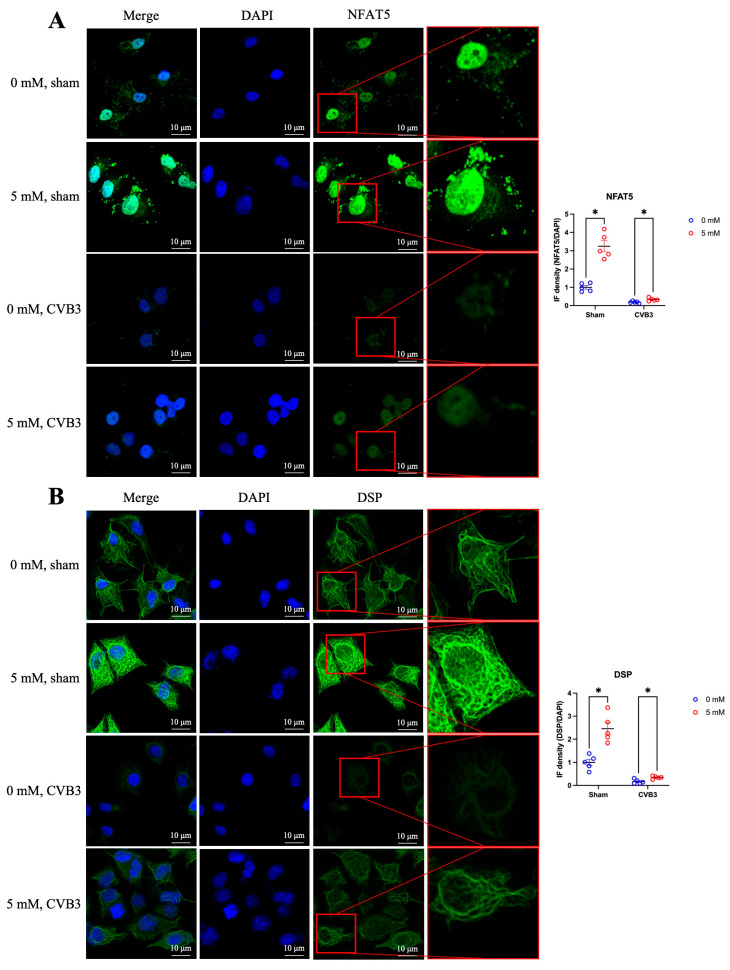

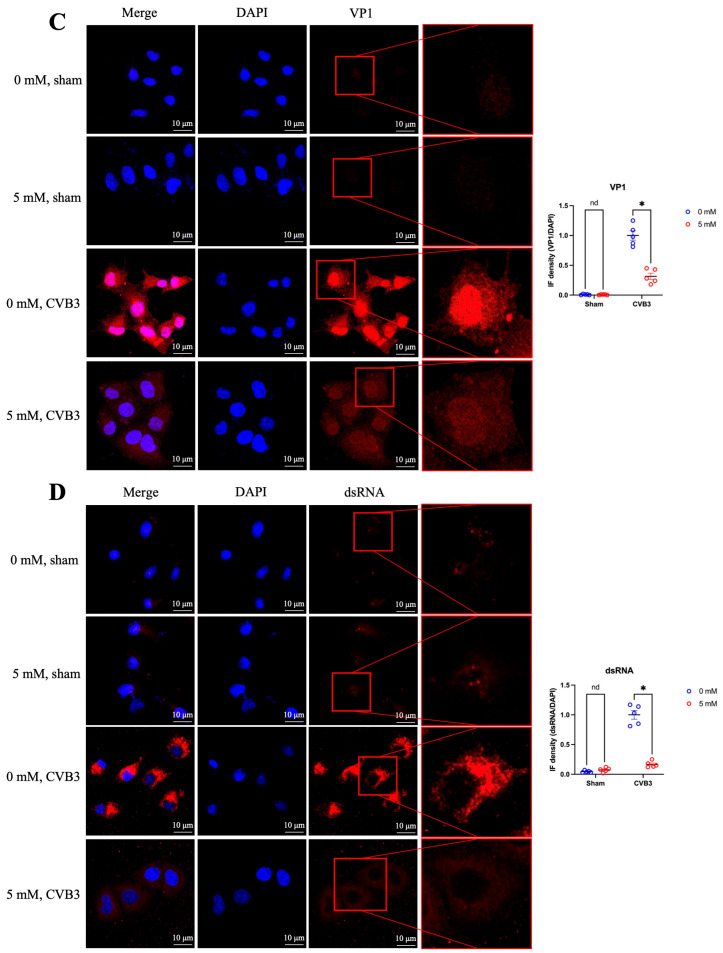
IF to determine the HPβCD-induced upregulation of both NFAT5 and desmoplakin, and suppression of CVB3 replication. Virus- and sham-infected HeLa cells were treated with 0 mM or 5 mM HPβCD for 24 h, followed by IF to evaluate the expression of (**A**) NFAT5, (**B**) desmoplakin, (**C**) CVB3 VP1, and (**D**) CVB3 dsRNA. Green fluorescence indicates NFAT5 or desmoplakin, red fluorescence indicates VP1 or dsRNA, and blue fluorescence marks the nuclei. Quantification of immunofluorescence density was performed by ImageJ. Statistical analysis was performed as in Figure 1. Data are presented as mean ± SEM, *n* = 5. * *p* < 0.05; nd indicates not significant.

NFAT5 deficiency has been shown to reduce desmoplakin expression, leading to the destruction of the desmosome complex. Given the robust NFAT5-associated antiviral effect of cyclodextrin mentioned above, we next examined whether cyclodextrin could restore desmoplakin levels by upregulating NFAT5 during CVB3 infection. We treated HeLa cells with a 5 mM solution of HPβCD for 24 h, followed by infection with CVB3 (MOI = 10), or sham infection, for 4 h. As expected, HPβCD treatment increased NFAT5 localization in both the nucleus and cytoplasm in sham and CVB3 conditions (Figure 5A). Correspondingly, desmoplakin signal intensity was also elevated in both CVB3-infected and sham-infected cells, suggesting that HPβCD-induced NFAT5 upregulation enhances desmoplakin expression (Figure 5B). In addition, IF staining of the viral capsid protein VP1 showed a clear reduction in its expression following HPβCD treatment, indicating suppressed viral protein synthesis (Figure 5C). Similarly, double-stranded RNA (dsRNA), an intermediate product of viral genome replication, also showed decreased production in HPβCD-treated cells (Figure 5D). All these results suggest that cyclodextrin not only restores desmoplakin expression by upregulating NFAT5, but also inhibits CVB3 replication at both the genome replication and protein synthesis levels.

### 3.6. Cyclodextrin Supresses the Production of Selected Inflammatory Cytokines in CVB3 Infection

Elevated circulating cytokines have been reported in patients with heart failure, and excessive cytokines can depress myocardial contractility [20]. In CVB3 infection, the expression of interferon beta (IFN-β, gene *IFNB1*), tumor necrosis factor alpha (TNF-α, gene *TNF*), interleukin-1 beta (IL-1β, gene *IL1B*) and interleukin-8 (IL-8, gene *CXCL8*) are highly upregulated and correlate with myocarditis severity [21,22,23]. Briefly, IFN-β plays a protective role in CVB3 infection, whereas TNF-α, IL-1 β and IL-8 promote cardiac damage [22,24,25,26].

**Figure 6:**
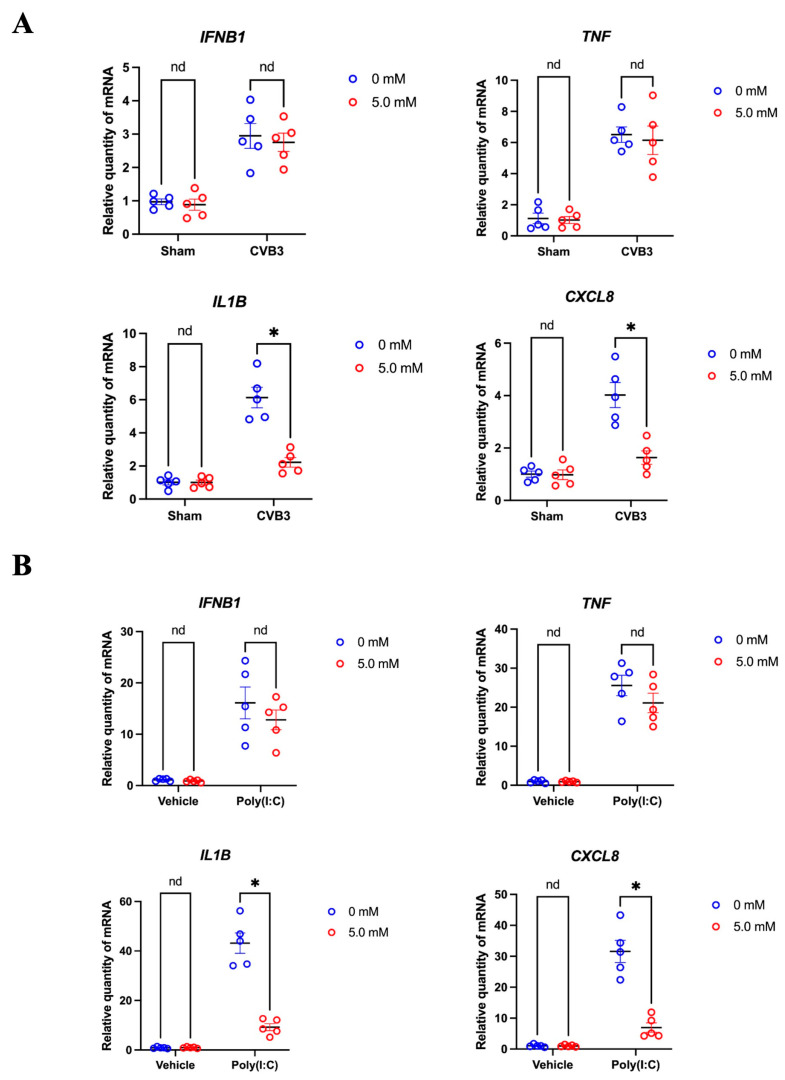
HPβCD regulates the mRNA levels of selected cytokines. HeLa cells were pretreated with 0 mM or 5 mM HPβCD for 24 h, followed by infection with CVB3 (MOI = 10) for 5 h (**A**) or transfection with Poly(I:C) for 6 h (**B**). Total RNA was extracted and subjected to RT-qPCR analysis to detect *IFNB1*, *TNF*, *IL1B,* and *CXCL8*. *GAPDH* was used as an internal control. Statistical analysis was performed using two-way ANOVA followed by Tukey’s test. Data are presented as mean ± SEM (*n* = 5). * *p* < 0.05; nd indicates not significant.

To assess the effect of cyclodextrin on cytokine responses during CVB3 infection, HeLa cells were pre-treated with a 5 mM solution of HPβCD for 24 h and infected with CVB3 (MOI = 10) for 5 h. By RT-qPCR, we found that cyclodextrin treatment did not significantly alter *IFNB1* and *TNF* transcriptional levels. Nevertheless, the mRNA levels of *IL1B* and *CXCL8* were significantly reduced in HPβCD-treated cells compared to the control (Figure 6A). Meanwhile, poly(I:C), a synthetic analog of virus double-stranded RNA (dsRNA), was used to mimic CVB3 RNA stimulation but avoid the effects of cleavage of many signal proteins in immune response pathway. Consistent with previous findings, cyclodextrin treatment reduced the mRNA levels of *IL1B* and *CXCL8*, but not *IFNB1* or *TNF* (Figure 6B). These results suggest that cyclodextrin may selectively suppress specific proinflammatory cytokines during CVB3 infection, potentially offering a targeted approach to mitigating excessive inflammatory responses.

## 4. Discussion

NFAT5 was previously identified by our group as a cardioprotective transcription factor in CVB3-infected mouse heart [10]. However, the mechanism of cardioprotection was not clear. This study, by introducing a potential NFAT5-inducing agent, cyclodextrin, reveals that NFAT5 protects cardiomyocytes from damage by maintaining the integrity of the desmosome, one of the three major complexes of the ICD. NFAT5 is an osmosensitive transcription factor upregulated during hypertonic stress conditions [27]. However, it is cleaved and inactivated by CVB3 proteases, leading to injury of the myocardium [9]. Thus, upregulating NFAT5 expression with identified hypertonic-inducing agents is a promising strategy to develop therapeutics for viral myocarditis. In this study, cyclodextrin, a cyclic oligosaccharide compound that is widely used in drug delivery and known for its ability to modulate cellular osmolality, emerges as an excellent candidate.

Desmosomes are essential for maintaining cardiac tissue integrity, with desmoplakin acting as a key component protein within the desmosomal complex. Our results show that knockout of *NFAT5* leads to a significant reduction in desmoplakin protein levels in the absence of CVB3 infection. This decrease was accompanied by desmosomal disassembly, as confirmed by TEM. These findings are consistent with previous reports linking desmosomal dysfunction to cardiomyopathies, including arrhythmogenic cardiomyopathy and viral myocarditis [2,3,28,29]. The observed loss of desmosomal integrity in the *Nfat5*-knockout suggests a previously unrecognized safeguarding role for this transcription factor in maintaining cardiac cell–cell adhesion and structural stability. Furthermore, we observed that CVB3 infection leads to a disruption of desmosomal complexes in mouse hearts. This finding is particularly relevant to the pathology of viral myocarditis, in which structural disintegration of the myocardium contributes to impaired cardiac function and arrhythmias.

In further investigation of the mechanism by which NFAT5 maintains the integrity of desmosome structure, we found, for the first time, that desmoplakin, a major component of the desmosome structure, is the direct transcription target of NFAT5. This discovery was supported by RT-qPCR analyses of *DSP* mRNA in both *Nfat5*-knockout mouse hearts and *Nfat5*-knockdown HeLa cells using specific siRNA. All these data suggest that NFAT5 maintains desmosome stability through induction of desmoplakin expression, thus suppressing its disassembly.

Cyclodextrins are highly water-soluble and can be administered directly without liposomal vectors. In preclinical and clinical studies, HPβCD has been delivered systemically by intravenous or oral routes, circulating freely in plasma. Because of their relatively small size (~1.3 kDa), cyclodextrins can distribute into extracellular spaces of multiple organs, including the heart. Animal studies have confirmed detectable levels of HPβCD in heart tissue after IV delivery [30]. Interestingly, cyclodextrin treatment upregulated NFAT5 expression and inhibited CVB3 replication in a dose-dependent manner, suggesting that rescue of NFAT5 by cyclodextrin treatment may contribute to enhanced antiviral defenses. To specifically assess the impact of cyclodextrin on intracellular viral replication, we first allowed CVB3 to infect cells for 1 h, ensuring viral entry, and subsequently refreshed the media (with or without cyclodextrin). This experimental design allowed us to focus the effects of cyclodextrin on post-viral entry processes. Our results revealed that cyclodextrin significantly inhibits CVB3 replication at multiple stages, including viral genome replication, viral protein synthesis, and virion assembly.

In addition to the above evidence that cyclodextrin suppresses CVB3 replication after viral entry, we also examined cytokines involved in virus-induced inflammatory responses. The life-threatening severity of myocarditis arises not only from viral replication in the heart but also from excessive production of proinflammatory cytokines during the acute phase of infection [31]. In our study, cyclodextrin did not significantly alter the transcriptional levels of IFN-β or TNF-α but did significantly reduce those of the proinflammatory cytokines IL-1β and IL-8. Because CVB3 proteases can cleave upstream pattern recognition receptors involved in IFN-β signaling, such as RIG-I and MDA5 [32,33], we also employed poly(I:C), an analog of viral dsRNA, to mimic viral stimulation of immune responses but avoid the effects of such cleavage. Consistently, cyclodextrin treatment selectively inhibited the production of IL-1β and IL-8 without broadly suppressing immune signaling via IFN-β and TNF-α. The mechanism underlying the selective suppression of cytokine expression merits further study, particularly using an in vivo model to fully understand the immunomodulatory effects of cyclodextrin. Taken together, our findings offer new insights into the therapeutic potential of cyclodextrin, not only for limiting viral replication but also for differentially modulating the inflammatory responses in CVB3-induced myocarditis.

In summary, this study identified NFAT5 as a key regulator of desmosomal stability and an integral component of the host defense against CVB3 infection, with desmoplakin confirmed as its direct transcriptional target. Loss of NFAT5 leads to desmoplakin downregulation and desmosomal disruption, which may underlie the cardiac dysfunction in viral myocarditis. More importantly, we identified cyclodextrin as a dual-functional agent counteracting CVB3-induced cardiac damages through NFAT5-DSP-mediated protection of desmosome integrity and suppression of proinflammatory cytokine expression. These results open new avenues for targeting NFAT5 with cyclodextrin in the treatment of viral myocarditis, and warrant in vivo evaluation to determine the efficacy and safety of this agent.

## Data Availability

The original contributions presented in this study are included in the article/Supplementary Material. Further inquiries can be directed to the corresponding author.

## Supplementary Materials

The following supporting information can be downloaded at: https://www.mdpi.com/article/10.3390/microorganisms13102294/s1, Table S1. qPCR primers sequence used in this study; Figure S1. Prediction of *DSP* Gene Promoter Region Targeted by NFAT5.
