# Supplementary material for: Cyclodextrin Counteracts Coxsackievirus-Induced Cardiac Damage by Protecting Desmosome Integrity and Suppressing Proinflammatory Cytokine Expression
Source: Microorganisms. 2025 Oct 2;13(10):2294. doi: 10.3390/microorganisms13102294 (PMC12566390; doi:10.3390/microorganisms13102294)
Supplement: Supplementary file 1 [file microorganisms-13-02294-s001.zip › microorganisms-3875416-supplementary.pdf]

Supplementary Material

Table S1. qPCR primers sequence used in this study

PRIMER	SEQUENCE (5’-3’)
h-*GAPDH* (forward)	GTCTCCTCTGACTTCAACAGCG
h-*GAPDH* (reverse)	ACCACCCTGTTGCTGTAGCCAA
m-*Gapdh* (forward)	CATCACTGCCACCCAGAAGACTG
m-*Gapdh* (reverse)	ATGCCAGTGAGCTTCCCGTTCAG
h-*DSP* (forward)	TGACAGACCGCTGGCAAAGGAT
h-*DSP* (reverse)	GGCGTTTAGCATCATAGAGCCAC
m-*Dsp* (forward)	TACACCTCAGGGCTGGAAACTC
m-*Dsp* (reverse)	GTAGTCTCCAGACCTCGTAAGC
h-*IFNB1* (forward)	CTTGGATTCCTACAAAGAAGCAGC
h-*IFNB1* (reverse)	TCCTCCTTCTGGAACTGCTGCA
h-*TNF* (forward)	CTCTTCTGCCTGCTGCACTTTG
h-*TNF* (reverse)	ATGGGCTACAGGCTTGTCACTC
h-*IL1B* (forward)	CCACAGACCTTCCAGGAGAATG
h-*IL1B* (reverse)	GTGCAGTTCAGTGATCGTACAGG
h-*CXCL8* (forward)	GAGAGTGATTGAGAGTGGACCAC
h-*CXCL8* (reverse)	CACAACCCTCTGCACCCAGTTT

Fig. S1

A

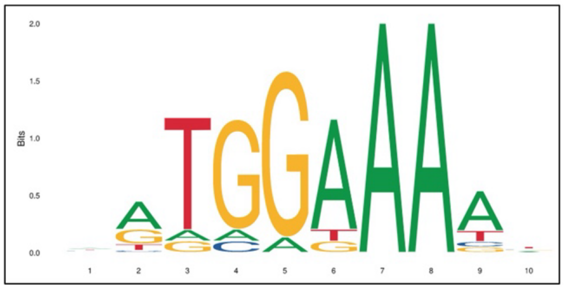

B

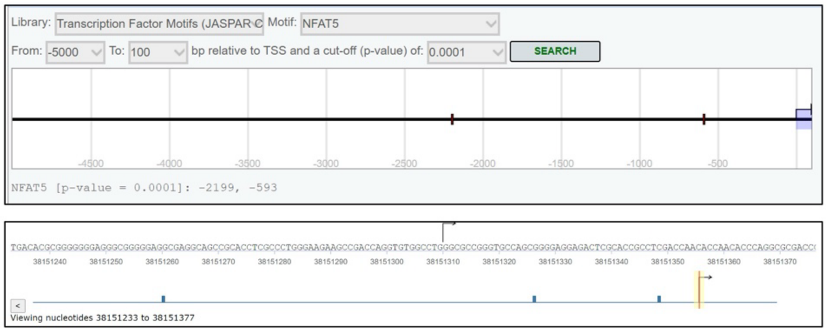

TSS@ 38151310, NFAT5 motif@ 38150785 => -525

Motif ID	Alt ID	Sequence Name	Strand	Start	End	p-value	q-value	Matched Sequence
MA0606.1	NFAT5	FP018094	-	4404	4413	7.32e-06	0.0601	GTTTTCCATT
MA0606.1	NFAT5	FP018094	+	2797	2806	1.22e-05	0.0601	attttccatc

5000-4404=596
=> -596

Figure S1. Prediction of DSP Gene Promoter Region Targeted by NFAT5. Prediction of the NFAT5 target sites was made by screening the NFAT5 binding motif in the promoter region of DSP gene. The promoter region we selected is from 5000 base pair upstream of its transcription start site (TSS) to 1000 base pair downstream of its TSS. By comparing the overlapping predictions from multiple programs (Fimo, Transfac and CiiiDER), one potential target site (-596) was determined on the promoter region of DSP.
